# A General Three-Parameter Logistic Model With Time Effect

**DOI:** 10.3389/fpsyg.2020.01791

**Published:** 2020-08-05

**Authors:** Zhaoyuan Zhang, Jiwei Zhang, Jian Tao, Ningzhong Shi

**Affiliations:** Key Laboratory of Applied Statistics of MOE, School of Mathematics and Statistics, Northeast Normal University, Changchun, China

**Keywords:** Bayesian inference, deviance information criterion (DIC), item response theory (IRT), logarithm of the pseudomarginal likelihood (LPML), Markov chain Monte Carlo (MCMC), three-parameter logistic model

## Abstract

Within the framework of item response theory, a new and flexible general three-parameter logistic model with response time (G3PLT) is proposed. The advantage of this model is that it can combine time effect, ability, and item difficulty to influence the correct-response probability. In contrast to the traditional response time models used in educational psychology, the new model incorporates the influence of the time effect on the correct-response probability directly, rather than linking them through a hierarchical method via latent and speed parameters as in van der Linden's model. In addition, the Metropolis–Hastings within Gibbs sampling algorithm is employed to estimate the model parameters. Based on Markov chain Monte Carlo output, two Bayesian model assessment methods are used to assess the goodness of fit between models. Finally, two simulation studies and a real data analysis are performed to further illustrate the advantages of the new model over the traditional three-parameter logistic model.

## 1. Introduction and Motivation

Computerized assessment has become a widely accepted method of testing owing to the fact that the results produced by examinees can be quickly and accurately evaluated by virtue of the computational power that is now available. In addition, with the help of computer technology, the response times of examinees are easier to collect than in the case of traditional paper-and-pencil tests. The collected response times provide a valuable source of information on examinees and test items. For example, response times can be used to improve the accuracy of ability estimates (van der Linden, 2007; Klein Entink et al., 2009a; van der Linden and Glas, 2010; Wang et al., 2013, 2018a; Wang and Xu, 2015; Fox and Marianti, 2016; Bolsinova and Tijmstra, 2018; De Boeck and Jeon, 2019), to detect rapid guessing and cheating behavior (van der Linden and Guo, 2008; van der Linden, 2009; Wang and Xu, 2015; Pokropek, 2016; Qian et al., 2016; Skorupski and Wainer, 2017; Wang et al., 2018a,b; Lu et al., 2019; Sinharay and Johnson, 2019; Zopluoglu, 2019), to evaluate the speededness of tests (Schnipke and Scrams, 1997; van der Linden et al., 2007), and to design more efficient tests (Bridgeman and Cline, 2004; Chang, 2004; Choe et al., 2018).

### 1.1. Advantages of Our Model Over Traditional Response Time Models in Educational Psychology Research

Although response times in both educational and psychological research have been studied widely and in depth, there are still some deficiencies in the existing literature. Here, we compare existing response time models with our new model and analyze the advantages of our model from multiple aspects.

**Figure 1:**
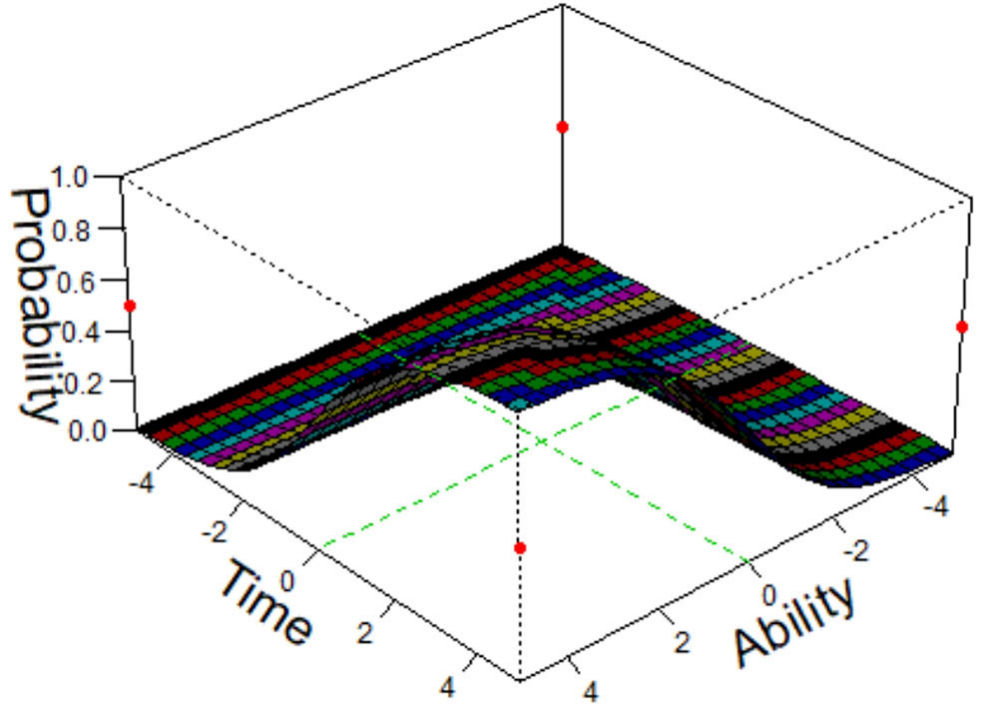
The 3-D diagram of ability, time, and correct response probability.

Thissen (1983) proposed a joint model of response time and accuracy to describe the speed-accuracy relationship. In his model, the speed-accuracy trade-off is reflected by letting response accuracy depend on the time devoted to an item: spending more time on an item increases the probability of a correct response. Thissen's joint model can be expressed as follows:

$$\log T_{ij}=u+\eta_{i}+\varsigma_{j}-\rho \left(a_{j}\theta_{i}-b_{j}\right)+\varepsilon_{ij},$$

where *T*_*ij*_ is the response time of the *i*th examinee answering the *j*th item, *u* is a general intercept parameter, η_*i*_ and ς_*j*_ can be interpreted, respectively as the speed of examinee *i* and the amount of time required by item *j*, ρ is a regression parameter, *a*_*j*_ and *b*_*j*_ are, respectively the item discrimination and difficulty parameters, θ_*i*_ is the ability parameter for the *i*th examinee, and $\varepsilon_{ij}\sim N\left(0,\sigma^{2}\right)$. The speed–accuracy trade–off is represented by the term *a*_*j*_θ_*i*_ − *b*_*j*_ when ρ < 0. When ρ > 0, the speed-accuracy relation is reversed. However, the way in which this model incorporates personal-level and item-level parameters means that it is unable to fully reflect the direct impact of the response time on the correct-response probability. Our new model solves this problem. The response time and the ability and item difficulty parameters are combined in an item response model that reflects the way in which the interactions among the three factors influences the correct-response probability. To provide an intuitive explanation, we use a three-dimensional diagram (Figure 1) to illustrate the effect of the ability and response time on the correct-response probability. A similar modeling method was proposed by Verhelst et al. (1997).

Roskam (1987, 1997) proposed a Rasch response time model integrating response time and correctness. According to this model, the probability of a correct response for the *i*th examinee answering the *j*th item can be written as

$$p\left(Y_{ij}=1|T_{ij},i,j\right)=\frac{\theta_{i}T_{ij}}{\theta_{i}T_{ij}+\delta_{j}}=\frac{\text{exp}\left(\xi_{i}+\tau_{ij}-\kappa_{j}\right)}{1+\text{exp}\left(\xi_{i}+\tau_{ij}-\kappa_{j}\right)},$$

where *Y*_*ij*_ denotes the response of the *i*th examinee answering the *j*th item, θ_*i*_ is the ability parameter for the *i*th examinee. δ_*j*_ is the item difficulty parameter for the *j*th item, and ξ_*i*_, τ_*ij*_, and κ_*j*_ are the logarithms of θ_*i*_, *T*_*ij*_, and δ_*j*_, respectively. We can see that when *T*_*ij*_ goes to infinity, the correct-response probability *p*(*Y*_*ij*_ = 1 ∣ *T*_*ij*_, *i, j*) approaches 1, no matter how difficult the item is. In fact, this type of model can only be applied to speeded tests, because a basic characteristic of such tests is that test items are quite easy, so, with unlimited time available, the answers are almost always correct. However, our new model is designed for a power test. This means that even if the examinees are given enough time, they cannot be sure to answer an item correctly, but rather they answer the item correctly with the probability of a three-parameter logistic (3PL) model.

Although there is some similarity between our model and the item response model proposed by Wang and Hanson (2005) with regard to the incorporation of response time into the traditional 3PL model, there are some major differences in concept and construction. Wang and Hanson give the probability of a correct response to item *j* by examinee *i* as

$$\begin{array}{l} \;p\left(Y_{ij}=1|a_{j},b_{j},c_{j},d_{j},\theta_{i},\eta_{i},T_{ij}\right)=c_{j}\\ +\frac{1-c_{j}}{1+\text{exp}\left[-1.7a_{j}\left(\theta_{i}-b_{j}-\eta_{i}d_{j}/T_{ij}\right)\right]}, \end{array}$$

where *a*_*j*_, *b*_*j*_, and *c*_*j*_ are, respectively the item discrimination, difficulty, and guessing parameters for the *j*th item, as in the regular 3PL model. θ_*i*_ and η_*i*_ are, respectively the ability and slowness parameters for the *i*th examinee, and *d*_*j*_ is the slowness parameter for the *j*th item. The item and personal slowness parameters determine the rate of increase in the probability of a correct answer as a function of response time. We will now analyze the differences between the two models.

From the perspective of model construction, the response time and the item and personal parameters are all incorporated into the same exponential function in Wang and Hanson's model, namely, exp[−1.7*a*_*j*_(θ_*i*_ − *b*_*j*_ − η_*i*_*d*_*j*_/*T*_*ij*_)], whereas in our model, the parameters and time effect appear in two different exponential functions (see the following section for a detailed description of the model): $\text{exp}\left[-1.7a_{j}\left(\theta_{i}-b_{j}\right)\right]+\text{exp}\left(-t_{ij}^{*}\right)$. Our model considers not only the influence of the personal and item factors on the correct-response probability, but also that of the time effect. In Wang and Hanson's model, two slowness parameters associated with persons and items are introduced on the basis of the traditional 3PL model, which increases the complexity of the model. The model can be identified only by imposing stronger constraints on the model parameters. The accuracy of parameter estimation may be reduced owing to the increase in the number of model parameters. However, in our model, no such additional parameters related to items and persons are introduced, and therefore the model is more concise and easy to understand. In terms of model identifiability, our model is similar to the traditional 3PL model in that no additional restrictions need to be imposed. More importantly, parameter estimation becomes more accurate because of the addition of time information. Besides the personal ability parameter, a personal slowness parameter is included Wang and Hanson's model. In fact, their model is a multidimensional item response theory model incorporating response time. In their model, it is assumed that these two personal parameters are independent, but this assumption may not necessarily be true in practice. For example, the lower a person's ability, the slower is their response. That is to say, there is a negative correlation between the ability parameter and the slowness parameter. More research is needed to verify this. Like other models based on the traditional 3PL model (see the next subsection), Wang and Hanson's model cannot distinguish between different abilities under different time intensities when examinees have the same response framework. However, our new model can deal with this problem very well.

In addition, our model introduces the concept of a time weight. Depending on the importance of a test (e.g., whether it is a high-stakes or a low-stakes test), the effect of the time constraint on the whole test is characterized by a time weight. This is something that cannot be dealt with by Wang and Hanson's model.

van der Linden (2007) proposed a hierarchical framework in which responses and response times are modeled separately at the measurement model level, while at a higher level, the ability and speed parameters are included in a population model to account for the correlation between them. In his approach, the latent speed parameter directly affects the response time, while the speed parameters and ability parameters are linked by the hierarchical model. It is known that in item response theory models, ability has a direct impact on the correct-response probability. Thus, we can see that the correct-response probability is related to the response time via the personal parameters (speed and ability). Van der Linden's hierarchical modeling method is unrealistic in that it includes the response time and the ability parameters in the item response model, whereas our model represents the relationships among response time, ability, and correct-response probability more simply and directly. Several other models have a similar structure to van der Linden's hierarchical model, including those of Fox et al. (2007), Klein Entink et al. (2009a,b), van der Linden and Glas (2010), Marianti et al. (2014), Wang and Xu (2015), Wang et al. (2018a), Fox and Marianti (2016), and Lu et al. (2019).

### 1.2. Advantages of Our Model Compared With the Traditional 3PL Model

Item response theory (IRT) models have been extensively used in educational testing and psychological measurement (Lord and Novick, 1968; van der Linden and Hambleton, 1997; Embretson and Reise, 2000; Baker and Kim, 2004). The most popular IRT model that includes guessing is the 3PL model (Birnbaum, 1968), which has been discussed in many papers and books (see e.g., Hambleton et al., 1991; van der Linden and Hambleton, 1997; Baker and Kim, 2004; von Davier, 2009; Han, 2012). However, several studies have revealed that the 3PL model has technical and theoretical limitations (Swaminathan and Gifford, 1979; Zhu et al., 2018). In this paper, we focus on another defect of the traditional 3PL model, namely, that it cannot distinguish between different abilities under different time intensities when the examinees have the same response framework. Here, we give a simulation example to illustrate the shortcomings of the traditional 3PL model and the advantages of our model (which is a general three-parameter logistic model with response time: G3PLT). We assume that 24 examinees answer three items and that the examinees can be divided into three groups of eight, with the examinees in each group having response frameworks (1, 0, 0), (0, 1, 0), and (1, 1, 0), respectively. Here, 0 indicates that the item is answered correctly and 1 indicating that it is answered incorrectly. The item parameters of the three items are calibrated in advance and known. The discrimination, difficulty, and guessing parameters are set as in Table 1.

**Table 1:** The setting of the true values of discrimination, difficulty, and guessing parameters.

**Item**	**Discrimination**	**Difficulty**	**Guessing**
1	0.8	−1	0
2	1	0	0.05
3	1.2	1	0.1

To consider the influence of different time effects on the ability of the examinees, eight time transformation values are considered: −0.2, 0.2, 0.5, 1, 2, 3, and 8. The specific settings for the time transformation values can be found in section 2. Table 2 shows the estimated ability values from the 3PL model and from our model under different response frameworks, with the maximum likelihood method being used to estimate the ability parameter.

**Table 2:** The comparisons of ability estimates under the frameworks of 3PL model and G3PLT model.

	**Fitting**	**Response**	**Transformated**	**Estimation of**
**Examinees**	**model**	**framework**	time t^*^	**ability**
1		(1, 0, 0)	−0.2	−0.8863
2		(1, 0, 0)	0	−0.8970
3		(1, 0, 0)	0.2	−0.9052
4	G3PLT	(1, 0, 0)	0.5	−0.9142
5		(1, 0, 0)	1	−0.9232
6		(1, 0, 0)	2	−0.9305
7		(1, 0, 0)	3	−0.9327
8		(1, 0, 0)	8	−0.9339
−	3PL	(1, 0, 0)	−	−0.9339
9		(0, 1, 0)	−0.2	0.1408
10		(0, 1, 0)	0	0.0614
11		(0, 1, 0)	0.2	−0.0139
12	G3PLT	(0, 1, 0)	0.5	−0.1233
13		(0, 1, 0)	1	−0.2945
14		(0, 1, 0)	2	−0.5397
15		(0, 1, 0)	3	−0.6515
16		(0, 1, 0)	8	−0.7202
−	3PL	(0, 1, 0)	−	−0.7207
17		(1, 1, 0)	−0.2	1.3109
18		(1, 1, 0)	0	1.0990
19		(1, 1, 0)	0.2	0.9791
20	G3PLT	(1, 1, 0)	0.5	0.8706
21		(1, 1, 0)	1	0.7752
22		(1, 1, 0)	2	0.7016
23		(1, 1, 0)	3	0.6785
24		(1, 1, 0)	8	0.6660
−	3PL	(1, 1, 0)	−	0.6659

The following conclusions can be drawn from Table 2.

1. The estimated ability under the G3PLT model with the same response framework will gradually increase as the transformed time decreases from 8 to −0.2. This indicates that the examinees may have different proficiencies in responding to items. Less time is taken if the examinee has greater ability. The time effect captures exactly the information that the traditional 3PL model cannot provide. Specifically, the 3PL model cannot distinguish between abilities when there are different response times under the same response framework.
2. As an illustration, we consider the case where the transformed time is −0.2. The ability estimates under the three response frameworks (1, 0, 0), (0, 1, 0), and (1, 1, 0) are −0.8863, 0.1408, and 1.3109, respectively. We find that the more difficult the item and the greater the number of items answered correctly, the higher are the ability estimates. Without considering the time effect, the ability estimates based on the 3PL model under the three response frameworks are −0.9339, −0.7207, and 0.6659, respectively.
3. Under the three response frameworks, the ability estimates obtained from the G3PLT model and the 3PL model are almost the same when the transformed time reaches 8. This indicates that even if the examinees are allowed enough time, they cannot be certain of answering an item correctly, but can do so only with the correct-response probability given by the 3PL model.

**Figure 2:**
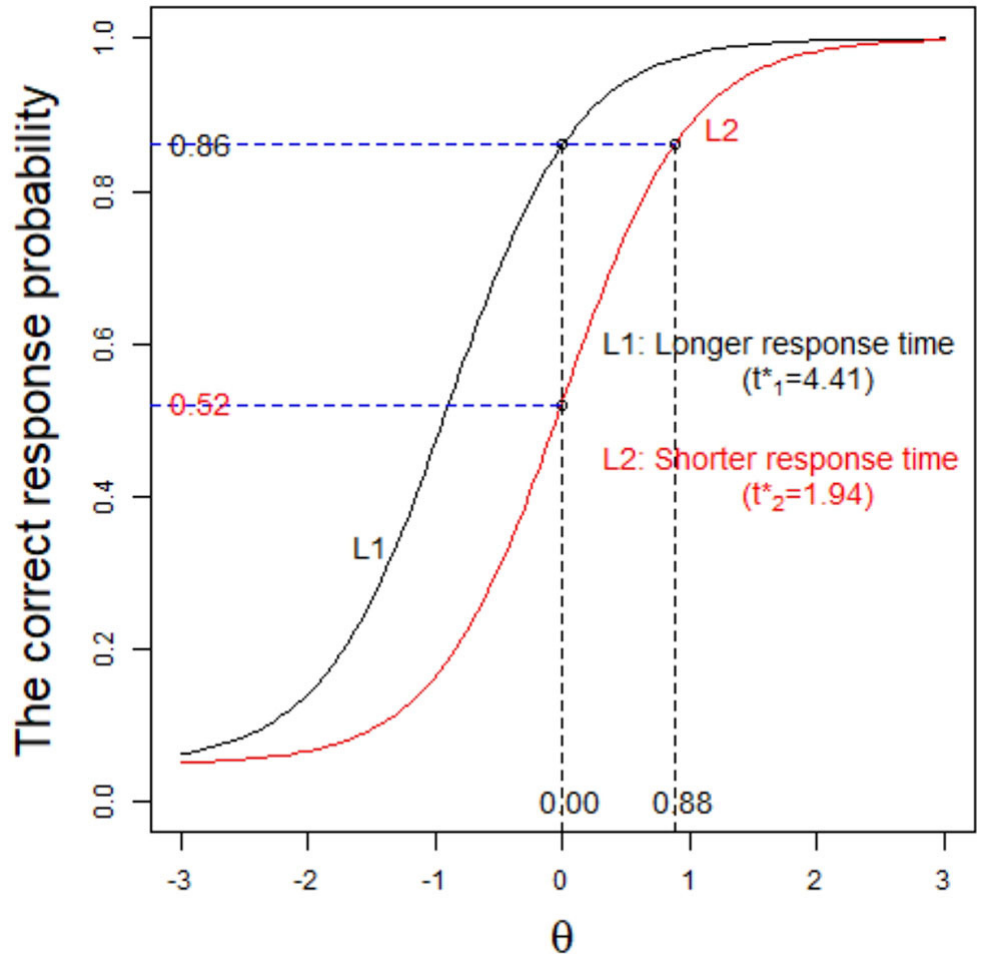
The item characteristic curve based on different time intensities.

We now give another example to further explain the advantages of the G3PLT model. Under the condition that the correct-response probability is the same, we consider the response times of examinees *i* and *j* when they answer the same item, and we find that these are 1 and 2 min, respectively. In general, we think that the examinee with shorter response times has a higher ability. Thus, here the ability of examinee *i* should be higher than that of examinee *j*. However, since the 3PL model does not consider response time, the difference in ability cannot be distinguished. This problem can be solved by using the G3PLT model. Because this model takes into account the information provided by response time, it can estimate the ability of examinees more objectively and accurately. As shown in Figure 2, for the same item, L1 represents the item characteristic curve corresponding to the case where examinees need a long response time ($t_{1}^{*}=4.41$), and L2 represents the item characteristic curve corresponding to the case where examinees need a short response time ($t_{1}^{*}=1.94$). When *p* = 0.86 is given as the correct-response probability, the estimated ability under L1 is 0, while the estimated ability under L2 is 0.88. Therefore, according to the evaluation results from the G3PLT model, the examinees with shorter times should have higher abilities, whereas the 3PL model is unable to distinguish between the two cases. In addition, it can be seen from the figure that when the ability is fixed at 0, the probabilities of a correct response under the two characteristic curves L1 and L2 are 0.86 and 0.52, respectively. This indicates that under the same ability condition, the correct-response probability of the examinees with short response times is lower than that of the examinees with long response times.

The remainder of this paper is organized as follows. Section 2 presents a detailed introduction to the proposed G3PLT model. Section 3 provides a computational strategy based on a Metropolis–Hastings within Gibbs sampling algorithm to meet computational challenges for the proposed model. Two Bayesian model comparison criteria are also discussed in section 3. In section 4, simulation studies are conducted to examine the performance of parameter recovery using the Bayesian algorithm and to assess model fit using the deviance information criterion (DIC) and the logarithm of the pseudomarginal likelihood (LPML). A real data analysis based on the Program for International Student Assessment (PISA) is presented in section 5. We conclude with a brief discussion and suggestions for further research in section 6.

## 2. The Model and Its Identification

### 2.1. The General Three-Parameter Logistic Model With Response Time (G3PLT)

Let the examinees be indexed by *i* = 1, 2, …, *N* and the items by *j* = 1, 2, …, *J*. Let θ denote the parameters representing the effects of the abilities of the examinees, and let *a*_*j*_, *b*_*j*_, and *c*_*j*_ denote the item effects, which are generally interpreted, respectively as discrimination power, difficulty, and success probability in the case of random guessing. If *Y*_*ij*_ denotes the response of the *i*th examinee answering the *j*th item, then the corresponding correct-response probability can be expressed as

(2.1) $$\begin{array}{l} p_{ij}=p\left(Y_{ij}=1|a_{j},b_{j},c_{j},\theta_{i},t_{ij}^{*}\right)\\ \;=c_{j}+\frac{1-c_{j}}{1+\text{exp}\left[-Da_{j}\left(\theta_{i}-b_{j}\right)\right]+\text{exp}\left(-t_{ij}^{*}\right)}, \end{array}$$

where *D* is a constant equal to 1.7. The influence of the time effect on the probability is described by the term $\text{exp}\left(-t_{ij}^{*}\right)$.

### 2.2. Time Transformation Function

It is obvious that when the response time of each item is very short, the correct-response probability of an item is reduced. In addition, we know that it is impossible for an examinee to answer an item 100% correctly even if they are given enough time to think about the item, and this can be attributed to limitations of the examinee's ability. When examinees are given enough time to answer each item, our model will reduce to the traditional 3PL model, and each item is answered correctly with the corresponding 3PL model correct-response probability. To make the model fully represent the requirement that the correct-response probability varies with time and to eliminate the effects of different average response times for each item in different tests, we consider the following time transformation:

(2.2) $$\begin{array}{l} t_{ij}^{*}=f\left(t_{ij}\right)=\frac{\log t_{ij}-\mu_{t}}{\sigma_{t}}+W, \end{array}$$

where μ_*t*_ is the logarithm of the average time spent by all examinees in answering all items, and σ_*t*_ is the corresponding standard deviation. *W* denotes the time weight, which is equal to zero or a positive integer. From the simulation study and real data analysis, we find that the G3PLT model reduces to the traditional 3PL model when the time weight increases to 8, and therefore we restrict the weight to values in the range 0–8. An increase in the time weight indicates that the time factor of the test has a small influence on the correct-response probability of the examinee.

Proposition 1. *Suppose that the correct-response probability* $p\left(Y_{ij}=1|a_{j},b_{j},c_{j},\theta_{i},t_{ij}^{*}\right)$ *is given by Equation (2.1). Then, we have the following results:*

1. *As the transformed time* $t_{ij}^{*}\to +\infty$, *the G3PLT model reduces to the 3PL model. That is,*
  (2.3) $$\begin{array}{l} p_{ij}\to c_{j}+\frac{1-c_{j}}{1+\text{ exp}\left[-Da_{j}\left(\theta_{i}-b_{j}\right)\right]}. \end{array}$$
  *In other words, it is impossible for the examinee to answer the item 100% correctly even if they are given enough time to think about the item, which can be attributed to the limitations of the examinee's ability*.
2. *As the transformed time* $t_{ij}^{*}\to -\infty$ *(the original time *t*_*ij*_ → 0), the correct-response probability of the G3PLT model tends to zero. That is,*
  (2.4) $$\begin{array}{l} p_{ij}=c_{j}+\frac{1-c_{j}}{1+\text{ exp}\left[-Da_{j}\left(\theta_{i}-b_{j}\right)+\text{ exp}\left(-t_{ij}^{*}\right)\right]}↓\;0. \end{array}$$
  *When there is not enough time to answer items (e.g., at the end of the examination), any item answered by the examinee must be one that requires only a very short time to finish. As the response time continues to shorten, the correct-response probability is reduced*.
3. *The G3PLT model can be reduced to a G2PLT model by constraining the lower asymptote parameter* *c*_*j*_ *to be zero, and a G1PLT model can be obtained by further constraining* *a*_*j*_ *to be the same across all items*.

### 2.3. Asymptotic Properties of the Model

Let *p*_*j*_ be the correct-response rate for the *j*th item. When the transformed time $t_{ij}^{*}\to +\infty$, the model in Equation (2.1) can be written as

(2.5) $$\begin{array}{l} \underset{t_{ij}^{*}\to +\infty }{\lim}\left\{c_{j}+\frac{1-c_{j}}{1+\text{ exp}\left[-Da_{j}\left(\theta_{i}-b_{j}\right)\right]+\text{ exp}\left(-t_{ij}^{*}\right)}\right\}\\ \;=c_{j}+\frac{1-c_{j}}{1+\text{ exp}\left[-Da_{j}\left(\theta_{i}-b_{j}\right)\right]}=p_{j}. \end{array}$$

The ability can be obtained as

(2.6) $$\begin{array}{l} \theta_{i}=b_{j}-\frac{1}{Da_{j}}\text{log}\left(\frac{1-p_{j}}{p_{j}-c_{j}}\right). \end{array}$$

**Figure 3:**
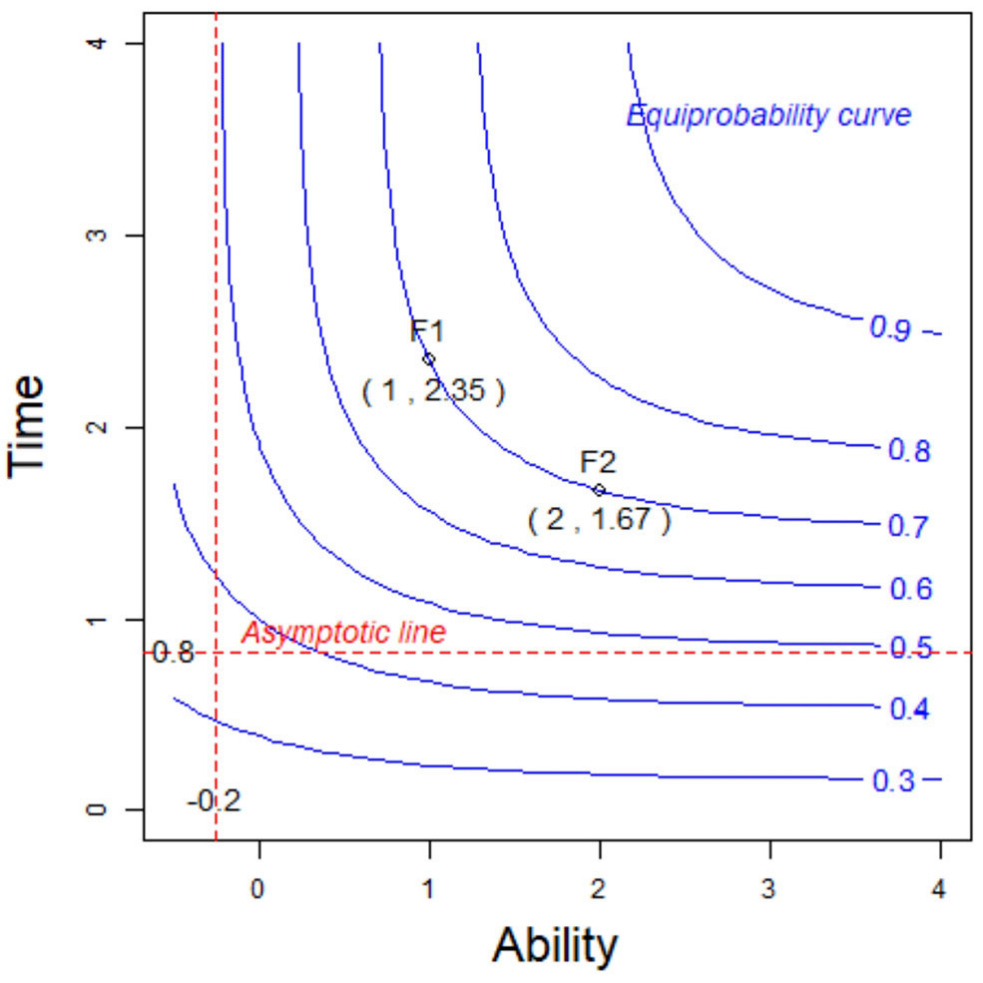
The equiprobability curve based on the ability and time.

Next, we will use a specific example to explain the meaning of Equations (2.5) and 2.6. Assuming that *p*_*j*_ = 0.5, *a*_*j*_ = 1.5, *b*_*j*_ = 1, and *c*_*j*_ = 0.1, we obtain θ_*i*_ = 0.8 from Equation (2.6). This result indicates that even if examinee *i* has sufficient response time to finish item *j*, the examinee's ability should be at least 0.8 (the intersection of the vertical asymptote and the *x*-axis in Figure 3) if the correct response probability reaches 0.5; otherwise, no matter how long a response time is allowed, the examinee's correct-response probability cannot reach 0.5. This is like a primary school pupil attempting to solve a college math problem, because the pupil's ability is so low that no matter how much time he is given, he cannot get a correct answer to item *j* other than by guessing. Moreover, when the ability θ_*i*_ → +∞, the model in Equation (2.1) can be written as

(2.7) $$\begin{array}{l} \underset{\theta_{i}\to +\infty }{\lim}\left\{c_{j}+\frac{1-c_{j}}{1+\text{ exp}\left[-Da_{j}\left(\theta_{i}-b_{j}\right)\right]+\text{ exp}\left(-t_{ij}^{*}\right)}\right\}\\ \;=c_{j}+\frac{1-c_{j}}{1+\text{ exp}\left(-t_{ij}^{*}\right)}=p_{j}. \end{array}$$

The transformed time $t_{ij}^{*}$ can be obtained as

(2.8) $$\begin{array}{l} t_{ij}^{*}=-\text{log}\left(\frac{1-p_{j}}{p_{j}-c_{j}}\right). \end{array}$$

**Figure 4:**
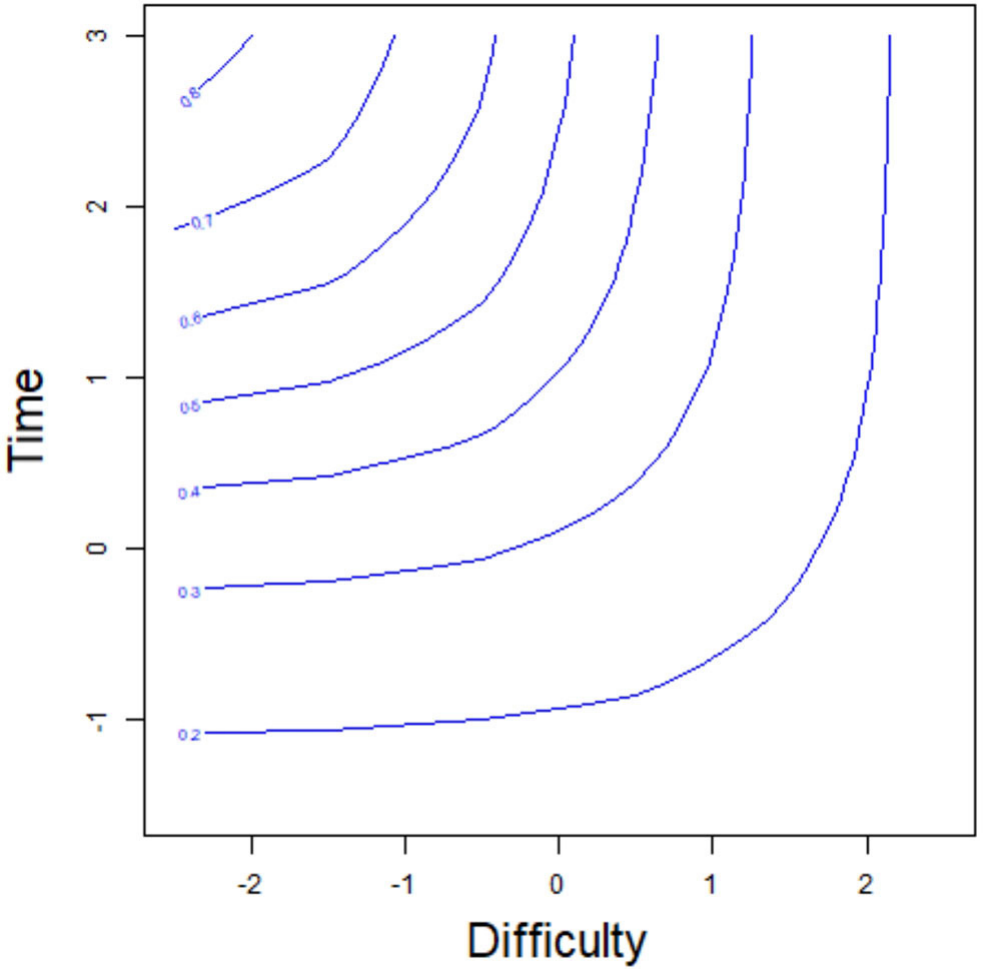
The equiprobability curve based on the item difficulty and time.

We again assume that *p*_*j*_ = 0.5, *a*_*j*_ = 1.5, *b*_*j*_ = 1, and *c*_*j*_ = 0.1. From (2.8), the transformed time $t_{ij}^{*}$ is about −0.2. This result indicates that even if the examinee *i* has a strong ability, the transformed time required to answer item *j* should not be less than −0.2 (the intersection of the horizontal asymptote and the *y*-axis in Figure 3) if the correct-response probability reaches 0.5; otherwise, no matter how strong the ability of the examinee, it is impossible to reach a correct-response probability of 0.5. This is like a college student solving a primary school math problem. Although the college student's ability is very strong, she cannot finish the item in a very short time. In addition, the correct-response probability of the examinees is the same for two points on the equiprobability curve. For example, for the two examinees F1 and F2 with the same correct-response probability 0.7 in Figure 3, the examinee F1 with low ability (1) takes a long time (2.35), while the response time (1.67) of the examinee F2 with high ability (2) is short to obtain the same correct-response probability. Similarly, the equiprobability curve based on item difficulty and time is shown in Figure 4. The correct-response probability is the same for two points on the equiprobability curve. The item with high difficulty takes a long time, while the response time of the item with low difficulty is short, giving the same correct-response probability.

### 2.4. Model Identification

To ensure identification of the G3PLT model, either the scale of latent traits or the scale of item parameters has to be restricted (Birnbaum, 1968; Lord, 1980; van der Linden and Hambleton, 1997). In this paper, we set the mean and variance of the latent traits to zero and one, respectively (Bock and Aitkin, 1981). The mean of the latent trait is fixed to remove the trade-off between θ_*i*_ and *b*_*j*_ in location, and the variance of the latent trait is fixed to remove the trade-off among θ_*i*_, *b*_*j*_, and *a*_*j*_ in scale.

## 3. Bayesian Inference

### 3.1. Prior and Posterior Distributions

In a Bayesian framework, the posterior distribution of the model parameters is obtained based on the observed data likelihood (sample information) and prior distributions (prior information). In general, these two kinds of information have an important influence on the posterior distribution. However, in large-scale educational assessment, the number of examinees is often very large. Therefore, the likelihood information plays a dominant role, and the selection of different priors (informative or non-informative) has no significant influence on the posterior inference (van der Linden, 2007; Wang et al., 2018a). Based on previous results (Wang et al., 2018a), we adopt the informative prior distribution to analyze the following simulation studies and real data. The specific settings are as follows. For the latent ability, we assume a standardized normal prior, i.e., θ_*i*_ ~ *N*(0, 1) for *i* = 1, …, *N*. The prior distribution for the discrimination parameter *a*_*j*_ is a lognormal distribution, i.e., *a*_*j*_ ~ log *N*(0, 1) for *j* = 1, …, *J*. The prior distribution for the difficulty parameter *b*_*j*_ is a standardized normal distribution, i.e., *b*_*j*_ ~ *N*(0, 1) for *j* = 1, …, *J*. For the guessing parameter, we assume a Beta distribution, i.e., *c*_*j*_ ~ Beta(2, 10) for *j* = 1, …, *J*. Then, the joint posterior distribution of the parameters given the data is as follows:

(3.1) $$\begin{array}{l} p\left(\theta ,a,b,c|Y,T\right)\propto \left[\overset{N}{\underset{i=1}{\prod }}\overset{J}{\underset{j=1}{\prod }}p\left(Y_{ij}|\theta_{i},a_{j},b_{j},c_{j},T_{ij}\right)\right]\overset{N}{\underset{i=1}{\prod }}p\left(\theta_{i}\right)\\ \;\times \overset{J}{\underset{j=1}{\prod }}p\left(a_{j}\right)p\left(b\right)p\left(c_{j}\right). \end{array}$$

### 3.2. Bayesian Estimation

Bayesian methods have been widely applied to estimate parameters in complex IRT models (see e.g., Albert, 1992; Patz and Junker, 1999a,b; Béguin and Glas, 2001; Rupp et al., 2004). In this study, the Metropolis-Hastings within Gibbs algorithm (Metropolis et al., 1953; Hastings, 1970; Tierney, 1994; Chib and Greenberg, 1995; Chen et al., 2000) is used to draw samples from the full conditional posterior distributions because the parameters of interest do not have conjugate priors within the framework of the IRT model.

#### Detailed MCMC Sampling Process

**Step 1**: Sample the ability parameter θ_*i*_ for the *i*th examinee. We independently draw $\theta_{i}^{*}$ from the normal proposal distribution, i.e., $\theta_{i}^{*}\sim N\left(\theta_{i}^{\left(r-1\right)},v_{\theta }^{2}\right)$. The prior of θ_*i*_ is assumed to follow a normal distribution with mean μ_θ_ and variance $\sigma_{\theta }^{2}$, i.e., $\theta_{i}\sim N\left(\mu_{\theta },\sigma_{\theta }^{2}\right)$. Therefore, the acceptance probability is given by

(3.2) $$\begin{array}{l} \alpha \left(\theta_{i}^{\left(r-1\right)},\theta_{i}^{*}\right)\\ =\min\left\{1,\frac{p\left(Y_{i}|\theta_{i}^{*},a^{\left(r-1\right)},b^{\left(r-1\right)},c^{\left(r-1\right)},T_{i}\right)p_{\text{prior}}\left(\theta_{i}^{*}|\mu_{\theta },\sigma_{\theta }^{2}\right)}{p\left(Y_{i}|\theta_{i}^{\left(r-1\right)},a^{\left(r-1\right)},b^{\left(r-1\right)},c^{\left(r-1\right)},T_{i}\right)p_{\text{prior}}\left(\theta_{i}^{\left(r-1\right)}|\mu_{\theta },\sigma_{\theta }^{2}\right)}\right\}. \end{array}$$

Otherwise, the value of the preceding iteration is retained, i.e., $\theta_{i}=\theta_{i}^{\left(r-1\right)}$. Here, ***Y***_*i*_ = (*Y*_*i*1_, *Y*_*ti*2_, …, *Y*_*iJ*_), ***T***_*i*_ = (*Y*_*i*1_, *Y*_*ti*2_, …, *Y*_*iJ*_), ***a*** = (*a*_1_, *a*_2_, …, *a*_*J*_), ***b*** = (*b*_1_, *b*_2_, …, *b*_*J*_), and ***c*** = (*c*_1_, *c*_2_, …, *c*_*J*_). In Equation (3.3), $p\left(Y_{i}|\theta_{i},a,b,T_{i}\right)=\prod_{j=1}^{J}{\left(p_{ij}\right)}^{y_{ij}}{\left(1-p_{ij}\right)}^{1-y_{ij}}$, where *p*_*ij*_ is given in Equation (2.1).

**Step 2**: Sample the difficulty parameter *b*_*j*_ for the *j*th item. We independently draw $b_{j}^{*}$ from the normal proposal distribution, i.e., $b_{j}^{*}\sim N\left(b_{j}^{\left(r-1\right)},v_{j}^{2}\right)$. The prior of *b*_*j*_ is assumed to follow a normal distribution with mean μ_*b*_ and variance $\sigma_{b}^{2}$, i.e., $b_{j}\sim N\left(\mu_{b},\sigma_{b}^{2}\right)$. The acceptance probability is given by

(3.3) $$\begin{array}{l} \alpha \left(b_{j}^{\left(r-1\right)},b_{j}^{*}\right)\\ =\min\left\{1,\frac{p\left(Y_{j}|\theta^{\left(r\right)},a_{j}^{\left(r-1\right)},b_{j}^{*},c_{j}^{\left(r-1\right)},T_{j}\right)p_{\text{prior}}\left(b_{j}^{*}|\mu_{b},\sigma_{b}^{2}\right)}{p\left(Y_{j}|\theta^{\left(r\right)},a_{j}^{\left(r-1\right)},b_{j}^{\left(r-1\right)},c_{j}^{\left(r-1\right)},T_{j}\right)p_{\text{prior}}\left(b_{j}^{\left(r-1\right)}|\mu_{b},\sigma_{b}^{2}\right)}\right\}. \end{array}$$

Otherwise, the value of the preceding iteration is retained, i.e., $b_{j}=b_{j}^{\left(r-1\right)}$. Here, ***Y***_*j*_ = (*Y*_1*j*_, *Y*_2*j*_, …, *Y*_*Nj*_), ***T***_*j*_ = (*T*_1*j*_, *T*_2*j*_, …, *T*_*Nj*_), and ***θ*** = (θ_1_, θ_2_, …, θ_*N*_). In Equation (3.3), $p\left(Y_{j}|\theta ,a_{j},b_{j},c_{j},T_{j}\right)=\prod_{i=1}^{n}{\left(p_{ij}\right)}^{y_{ij}}{\left(1-p_{ij}\right)}^{1-y_{ij}}$.

**Step 3**: Sample the discrimination parameter *a*_*j*_ for the *j*th item. We independently draw $a_{j}^{*}$ from the log-normal proposal distribution, i.e., $a_{j}^{*}\sim \text{log }N\left(\text{log }a_{j}^{\left(r-1\right)},v_{a}^{2}\right)$. In addition, *p*_prior_(*a*_*j*_) is a lognormal prior distribution, i.e., $a_{j}\sim \text{log }N\left(\mu_{a},\sigma_{a}^{2}\right)$. The acceptance probability is given by

(3.4) $$\begin{array}{l} \alpha \left(a_{j}^{\left(r-1\right)},a_{j}^{*}\right)\\ =\min\left\{1,\frac{p\left(Y_{j}|\theta^{\left(r\right)},a_{j}^{*},b_{j}^{\left(r\right)},c_{j}^{\left(r-1\right)},T_{j}\right)p_{\text{prior}}\left(a_{j}^{*}|\mu_{a},\sigma_{a}^{2}\right)a_{j}^{*}}{p\left(Y_{j}|\theta^{\left(r\right)},a_{j}^{\left(r-1\right)},b_{j}^{\left(r\right)},c_{j}^{\left(r-1\right)},T_{j}\right)p_{\text{prior}}\left(a_{j}^{\left(r-1\right)}|\mu_{a},\sigma_{a}^{2}\right)a_{j}^{\left(r-1\right)}}\right\}. \end{array}$$

Otherwise, the value of the preceding iteration is retained, i.e., $a_{j}=a_{j}^{\left(r\right)}$. In Equation (3.4), $\left(Y_{j}|\theta ,a_{j},b_{j},c_{j},T_{j}\right)=\prod_{i=1}^{n}{\left(p_{ij}\right)}^{y_{ij}}{\left(1-p_{ij}\right)}^{1-y_{ij}}$.

**Step 4**: Sample the guessing parameter *c*_*j*_ for the *j*th item. We independently draw $c_{j}^{*}$ from the uniform proposal distribution, i.e., $c_{j}^{*}\sim U\left(c_{j}^{\left(r-1\right)}-0.01,c_{j}^{\left(r-1\right)}+0.01\right)$. The prior of *c*_*j*_ is assumed to follow a Beta distribution, i.e., *c*_*j*_ ~ Beta(υ_1_, υ_2_). Therefore, the acceptance probability is given by

(3.5) $$\begin{array}{l} \alpha \left(c_{j}^{\left(r-1\right)},c_{j}^{*}\right)\\ =\min\left\{1,\frac{p\left(Y_{j}|\theta^{\left(r\right)},a_{j}^{\left(r\right)},b_{j}^{\left(r\right)},c_{j}^{*},T_{j}\right)p_{\text{prior}}\left(c_{j}^{*}|\upsilon_{1},\upsilon_{2}\right)}{p\left(Y_{j}|\theta^{\left(r\right)},a_{j}^{\left(r\right)},b_{j}^{\left(r\right)},c_{j}^{\left(r-1\right)},T_{j}\right)p_{\text{prior}}\left(c_{j}^{\left(r-1\right)}|\upsilon_{1},\upsilon_{2}\right)}\right\}. \end{array}$$

Otherwise, the value of the preceding iteration is retained, i.e., $c_{j}=c_{j}^{\left(r\right)}$. In Equation (3.5), $p\left(Y_{j}|\theta ,a_{j},b_{j},c_{j},T_{j}\right)=\prod_{i=1}^{n}{\left(p_{ij}\right)}^{y_{ij}}{\left(1-p_{ij}\right)}^{1-y_{ij}}$.

### 3.3. Bayesian Model Assessment

Spiegelhalter et al. (2002) proposed the deviance information criterion (DIC) for model comparison when the number of parameters is not clearly defined. The DIC is an integrated measure of model fit and complexity. It is defined as the sum of a deviance measure and a penalty term for the effective number of parameters based on a measure of model complexity. We write **Ω** = (**Ω**_*ij*_, *i* = 1, …, *N, j* = 1, …, *J*), where $\Omega_{ij}={\left(\theta_{i},a_{j},b_{j},c_{j}\right)}^{\prime }$. Let {**Ω**^(1)^, …, **Ω**^(*R*)^}, where $\Omega^{\left(r\right)}=\left(\Omega_{ij}^{\left(r\right)},\;i=1,\ldots ,N,\;j=1,\ldots ,J\right)$, $\Omega_{ij}^{\left(r\right)}={\left(\theta_{i}^{\left(r\right)},a_{j}^{\left(r\right)},b_{j}^{\left(r\right)},c_{j}^{\left(r\right)}\right)}^{\prime }$ for *i* = 1, …, *N*, *j* = 1, …, *J*, and *r* = 1, …, *R*, denote an Markov chain Monte Carlo (MCMC) sample from the posterior distribution in Equation (3.1). The joint likelihood function of the responses can be written as

(3.6) $$\begin{array}{l} L\left(Y|\Omega ,T\right)=\overset{N}{\underset{i=1}{\prod }}\overset{J}{\underset{j=1}{\prod }}f\left(y_{ij}|\theta_{i},a_{j},b_{j},c_{j},t_{ij}\right), \end{array}$$

where *f*(*y*_*ij*_ ∣ θ_*i*_, *a*_*j*_, *b*_*j*_, *c*_*j*_, *t*_*ij*_) is the response probability of the G3PLT model. The logarithm of the joint likelihood function in Equation (3.6) evaluated at **Ω**^(*r*)^ is given by

(3.7) $$\begin{array}{l} \text{log }L\left(Y|\Omega^{\left(r\right)},T\right)=\overset{N}{\underset{i=1}{\sum }}\overset{J}{\underset{j=1}{\sum }}\text{log }f\left(y_{ij}|\theta_{i}^{\left(r\right)},a_{j}^{\left(r\right)},b_{j}^{\left(r\right)},c_{j}^{\left(r\right)},t_{ij}\right). \end{array}$$

The joint log-likelihoods for the responses, $\text{log }f\left(y_{ij}|\theta_{i}^{\left(r\right)},a_{j}^{\left(r\right)},b_{j}^{\left(r\right)},c_{j}^{\left(r\right)},t_{ij}\right)$, *i* = 1, …, *N* and *j* = 1, …, *J*, are readily available from MCMC sampling outputs, and therefore $\text{log }f\left(y_{ij}|\theta_{i}^{\left(r\right)},a_{j}^{\left(r\right)},b_{j}^{\left(r\right)},c_{j}^{\left(r\right)},t_{ij}\right)$ in Equation (3.7) is easy to compute. The effective number of parameters in the models is defined by

(3.8) $$\begin{array}{l} p_{D}=\bar{\text{Dev}\left(\Omega \right)}-\text{Dev}\left(\hat{\Omega }\right), \end{array}$$

where $\bar{\text{Dev}\left(\Omega \right)}$ is a Monte Carlo estimate of the posterior expectation of the deviance function Dev(**Ω**) = −2 log *L*(***Y*** ∣ **Ω**, ***T***), and the term $\text{Dev}\left(\hat{\Omega }\right)$ is computed by plugging the mean of the simulated values of **Ω** into Dev(**·**), where $\hat{\Omega }=\overset{R}{\underset{r=1}{\sum }}\Omega^{\left(r\right)}/R$. More specifically,

(3.9) $$\begin{array}{l} \begin{array}{c} \bar{\text{Dev}\left(\Omega \right)}=-\frac{2}{R}\overset{R}{\underset{r=1}{\sum }}\text{log }L\left(Y|\Omega^{\left(r\right)}\right),\\ \text{Dev}\left(\hat{\Omega }\right)=-2\text{ log }L\left(Y|\hat{\Omega }\right). \end{array} \end{array}$$

The DIC can now be formulated as follows:

(3.10) $$\begin{array}{l} \text{DIC}=\hat{\text{Dev}\left(\Omega \right)}+2p_{D}=\hat{\text{Dev}\left(\Omega \right)}+2[\bar{\text{Dev}\left(\Omega \right)}-\hat{\text{Dev}\left(\Omega \right)}], \end{array}$$

A model with a smaller DIC value fits the data better.

Another method is to use the logarithm of the pseudomarginal likelihood (LPML) (Geisser and Eddy, 1979; Ibrahim et al., 2001) to compare different models. This is also based on the log-likelihood functions evaluated at the posterior samples of model parameters. The detailed calculation process is as follows.

We let $U_{ij,\max}=\max_{1\leq r\leq R}\left[-\text{log }f\left(y_{ij}|\theta_{i}^{\left(r\right)},a_{j}^{\left(r\right)},b_{j}^{\left(r\right)},c_{j}^{\left(r\right)},t_{ij}\right)\right]$, and a Monte Carlo estimate of the conditional predictive ordinate (CPO) (Gelfand et al., 1992; Chen et al., 2000) is then given by

(3.11) $$\begin{array}{l} \text{log}\hat{\left(\text{CP}{\text{O}}_{ij}\right)}=-U_{ij,\max}\\ -\text{log }\left\{\frac{1}{R}\overset{R}{\underset{r=1}{\sum }}\text{exp}\left[-\text{log}f\left(y_{ij}|\theta_{i}^{\left(r\right)},a_{j}^{\left(r\right)},b_{j}^{\left(r\right)},c_{j}^{\left(r\right)},t_{ij}\right)-U_{ij,\max}\right]\right\}. \end{array}$$

Note that the maximum value adjustment used in $\text{log}\hat{\left(\text{CP}{\text{O}}_{ij}\right)}$ plays an important role in numerical stabilization in the computation of $\text{exp}\left[-\text{log }f\left(y_{ij}|\theta_{i}^{\left(r\right)},a_{j}^{\left(r\right)},b_{j}^{\left(r\right)},c_{j}^{\left(r\right)},t_{ij}\right)-U_{ij,\max}\right]$ in Equation (3.11). A summary statistic of the $\hat{\text{CP}{\text{O}}_{ij}}$ is the sum of their logarithms, which is called the LPML and is given by

(3.12) $$\begin{array}{l} \text{LPML}=\overset{N}{\underset{i=1}{\sum }}\overset{J}{\underset{j=1}{\sum }}\text{log}\hat{\left(\text{CP}{\text{O}}_{ij}\right)}. \end{array}$$

A model with a larger LPML has a better fit to the data.

### 3.4. Accuracy Evaluation of Parameter Estimation

To implement the MCMC sampling algorithm, chains of length 10,000 with an initial burn-in period 5,000 are chosen. In the following simulation study, 200 replications are used. Five indices are used to assess the accuracy of the parameter estimates. Let ϑ be the parameter of interest. Assume that *M* = 200 data sets are generated. Also, let ${\hat{\vartheta }}^{\left(m\right)}$ and SD^(*m*)^(ϑ) denote the posterior mean and the posterior standard deviation of ϑ obtained from the *m*th simulated data set for *m* = 1, …, *M*.

The bias for the parameter ϑ is defined as

(3.13) $$\begin{array}{l} \text{Bias}\left(\vartheta \right)=\frac{1}{M}\overset{M}{\underset{m=1}{\sum }}\left({\hat{\vartheta }}^{\left(m\right)}-\vartheta \right), \end{array}$$

and the mean squared error (MSE) for ϑ is defined as

(3.14) $$\begin{array}{l} \text{MSE}\left(\vartheta \right)=\frac{1}{M}\overset{M}{\underset{m=1}{\sum }}{\left({\hat{\vartheta }}^{\left(m\right)}-\vartheta \right)}^{2}. \end{array}$$

The simulation SE is the square root of the sample variance of the posterior estimates over different simulated data sets. It is defined as

(3.15) $$\begin{array}{l} \text{Simulation SE}\left(\vartheta \right)=\sqrt{\frac{1}{M}\overset{M}{\underset{m=1}{\sum }}{\left({\hat{\vartheta }}^{\left(m\right)}-\frac{1}{M}\overset{M}{\underset{\ell =1}{\sum }}{\hat{\vartheta }}^{\left(\ell \right)}\right)}^{2}}, \end{array}$$

and the average of posterior standard deviation is defined as

(3.16) $$\begin{array}{l} \text{SD}\left(\vartheta \right)=\frac{1}{M}\overset{M}{\underset{m=1}{\sum }}\text{S}{\text{D}}^{\left(m\right)}\left(\vartheta \right). \end{array}$$

The coverage probability based on the 95% highest probability density (HPD) intervals is defined as

(3.17) $$\begin{array}{l} \text{CP}\left(\vartheta \right)\\ =\frac{\#\text{ of 95\% (HPD) intervals containing }\vartheta \text{ in }M\text{ simulated data sets }}{M}. \end{array}$$

## 4. Simulation Study

### 4.1. Simulation 1

We conduct a simulation study to evaluate the recovery performance of the combined MCMC sampling algorithm based on different simulation conditions.

#### Simulation Design

The following manipulated conditions are considered: (a) test length *J* = 20 or 60 and (b) number of examinees *N* = 500, 1, 000, or 2, 000. Fully crossing different levels of these two factors yields six conditions (two test lengths × three sample sizes). Next, the true values of the parameters are given. True item discrimination parameters *a*_*j*_ are generated from a truncated normal distribution, i.e., *a*_*j*_ ~ *N*(1, 0.2)I(*a*_*j*_ > 0), *j* = 1, 2, …, *N*, where the indicator function I(*A*) takes a value of 1 if *A* is true and a value of 0 if *A* is false. The item difficulty parameters *b*_*j*_ are generated from a standardized normal distribution. The item guessing parameters *c*_*j*_ are generated from a Beta distribution, i.e., *c*_*j*_ ~ Beta(2, 10). In addition, the ability parameters of the examinees, θ_*i*_, are also generated from a standardized normal distribution. In each simulation condition, 200 replications (replicas) are considered. Next, we generate the response time data for each examinee based on the following facts:

1. The difficulty of each item has a direct impact on the response time. That is to say, the time spent on simple items is shorter, and the time spent on difficult items is longer.
2. In addition, the ability of each examinee also has a direct impact on the response time. That is to say, examinees with higher ability spend less time on an item.
3. Depending on the importance of the test (high-stakes test or low-stakes test), the effect of the time constraint on the whole test should be characterized by the time weighting.

In Wang and Xu (2015, p. 459), the average logarithms of the response times for each item based on the solution behavior follow a normal distribution. That is, log *t*_*j*_ ~ *N*(0.5, 0.25), *j* = 1, 2, …, *J*, where the average time *t*_*j*_ spent on item *j* is about 1.64872 (= *e*^0.5^) min. We take the standardized transformation $t_{j}^{*}=f\left(t_{j}\right)=\left(\text{log }t_{j}-0.5\right)/0.5$, so that $t_{j}^{*}\sim N\left(0,1\right)$, where $-\infty <t_{j}^{*}<+\infty$.

Next, we consider the premise that the easier an item, the shorter is the response time. The true values of the difficulty parameter and the transformed time $t_{j}^{*}$ for each item are arranged in order from small to large, i.e., *b*_1_ < *b*_2_ < ⋯ < *b*_*J*−1_ < *b*_*J*_ and $t_{1}^{*}<t_{2}^{*}<\cdots <t_{J-1}^{*}<t_{J}^{*}$. The corresponding item–time pairs can be written as $\left(b_{1},t_{1}^{*}\right)<\left(b_{2},t_{2}^{*}\right)<\cdots <\left(b_{J-1},t_{J-1}^{*}\right)<\left(b_{J},t_{J}^{*}\right)$. The response time of each examinee is generated from a normal distribution, i.e., $t_{ij}^{*}\sim N\left(t_{j}^{*},0.5\right)$, where *j* = 1, …, *J*. Moreover, for a given item *j*, the premise that examinees with higher ability spend less time on the item needs to be satisfied. Therefore, we arrange θ_1*j*_ > θ_2*j*_ > ⋯ > θ_*N*−1, *j*_ > θ_*N, j*_, and $t_{1j}^{*}<t_{2j}^{*}<\cdots <t_{N-1,j}^{*}<t_{N,j}^{*}$. The corresponding ability–time pairs can be obtained by arranging the true values of the ability parameter and the transformed time $t_{ij}^{*}$, i.e., $\left(\theta_{ij},t_{ij}^{*}\right)$. The time weights range from 0 to 8. The higher the value of the time weight, the weaker is the influence of the time factor of the test on the correct-response probability of the examinee. In this simulation study, we assume that the time factor of the test has an important influence on the correct-response probability of the examinee. Therefore, we set the time weight to 1 in this simulation. Based on the true values of the parameters and the response time data, the response data can be simulated using the G3PLT model given by Equation (2.1).

#### Convergence Diagnostics

To evaluate the convergence of the parameter estimations, we only consider convergence in the case of minimum sample sizes. That is, the test length is fixed at 20, and the number of examinees is 500. Two methods are used to check the convergence of our algorithm. One is the “eyeball” method to monitor convergence by visually inspecting the history plots of the generated sequences (Zhang et al., 2007; Hung and Wang, 2012), and the other is the Gelman–Rubin method (Gelman and Rubin, 1992; Brooks and Gelman, 1998) for checking the convergence of the parameters.

**Figure 5:**
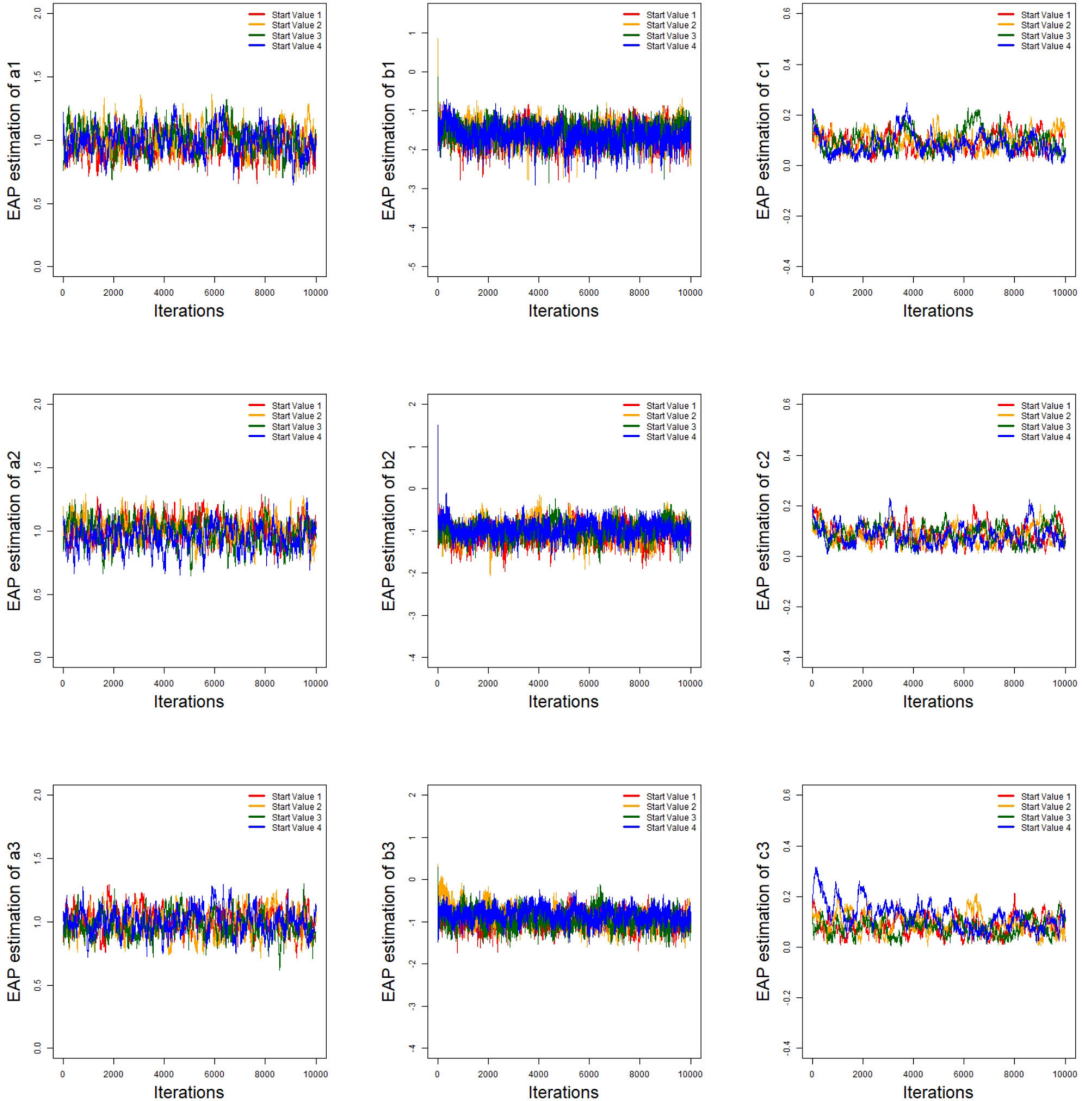
The trace plots of three randomly selected items for the simulation study 1.

The convergence of the Bayesian algorithm is checked by monitoring the trace plots of the parameters for consecutive sequences of 10,000 iterations. The trace plots show that all parameter estimates stabilize after 5,000 iterations and then converge quickly. Thus, we set the first 5,000 iterations as the burn-in period. As an illustration, four chains started at overdispersed starting values are run for each replication. The trace plots of three randomly selected items are shown in Figure 5. In addition, we find that the potential scale reduction factor (PSRF) (Brooks and Gelman, 1998) values for all parameters are less than 1.2, which ensures that all chains converge as expected.

#### Recovery of Item Parameters

The average bias, MSE, SD, SE, and CP for discrimination, difficulty, and guessing parameters based on six different simulation conditions are shown in Table 3. The following conclusions can be drawn.

1. Given the total test length, when the number of individuals increases from 500 to 2,000, the average MSE, SD, and SE for the discrimination, difficulty, and guessing parameters show a decreasing trend. For example, for a total test length of 20 items, when the number of examinees increases from 500 to 2,000, the average MSE of all discrimination parameters decreases from 0.0088 to 0.0072, the average SE of all discrimination parameters decreases from 0.0022 to 0.0014, and the average SD of all discrimination parameters decreases from 0.0085 to 0.0066. The average MSE of all difficulty parameters decreases from 0.0436 to 0.0213, the average SE of all difficulty parameters decreases from 0.0272 to 0.0122, and the average SD of all difficulty parameters decreases from 0.0362 to 0.0143. The average MSE of all guessing parameters decreases from 0.0019 to 0.0013, the average SE of all guessing parameters decreases from 0.0007 to 0.0006, and the average SD of all guessing parameters decreases from 0.0013 to 0.0008.
2. The average SDs of the item parameters are larger than their average SEs. This indicates that the fluctuations of the posterior means of item parameters between different replications are small compared with their fluctuations within each replication.
3. Under the six simulated conditions, the average CPs of the discrimination, difficulty, and guessing parameters are about 0.950.
4. When the number of examinees is held fixed but the number of items increases from 20 to 40, the average MSE, SD, and SE show that the recovery results for the discrimination, difficulty and guessing parameters do not change much, which indicates that the Bayesian algorithm is stable and there is no reduction in accuracy due to an increase in the number of items.

**Table 3:** Evaluating the accuracy of parameters based on six different simulated conditions in simulation study 1.

	**No. of items=20**														

	**No. of examinees 500**					**No. of examinees 1,000**					**No. of examinees 2,000**				
**Item parameter**	**Bias**	**MSE**	**SE**	**SD**	**CP**	**Bias**	**MSE**	**SE**	**SD**	**CP**	**Bias**	**MSE**	**SE**	**SD**	**CP**
Discrimination ***a***	−0.0162	0.0088	0.0022	0.0085	0.9513	−0.0081	0.0083	0.0019	0.0076	0.9503	−0.0038	0.0072	0.0014	0.0066	0.9480
Difficulty ***b***	−0.0134	0.0436	0.0272	0.0362	0.9385	−0.0103	0.0290	0.0166	0.0213	0.9410	−0.0068	0.0213	0.0122	0.0143	0.9287
Guessing ***c***	−0.0031	0.0019	0.0007	0.0013	0.9315	−0.0026	0.0016	0.0006	0.0010	0.9378	−0.0014	0.0013	0.0006	0.0008	0.9283
	**No. of items=60**														
	**No. of examinees 500**					**No. of examinees 1,000**					**No. of examinees 2,000**				
**Item parameter**	**Bias**	**MSE**	**SE**	**SD**	**CP**	**Bias**	**MSE**	**SE**	**SD**	**CP**	**Bias**	**MSE**	**SE**	**SD**	**CP**
Discrimination ***a***	0.0159	0.0082	0.0023	0.0082	0.9543	0.0132	0.0081	0.0019	0.0071	0.9393	0.0005	0.0074	0.0014	0.0059	0.9343
Difficulty ***b***	−0.0345	0.0447	0.0245	0.0339	0.9574	−0.0112	0.0233	0.0153	0.0205	0.9497	−0.0086	0.0163	0.0098	0.0121	0.9296
Guessing ***c***	0.0071	0.0016	0.0005	0.0011	0.9389	0.0061	0.0013	0.0005	0.0008	0.9328	0.0025	0.0011	0.0005	0.0006	0.9484

*The Bias, MSE, SD, SE, and CP denote the average; Bias, MSE, SE, SD, and CP for the parameters*.

a *represents all discrimination parameters*,

b *represents all difficulty parameters and **c** represents all guessing parameters*.

In summary, the Bayesian algorithm provides accurate estimates of the item parameters for various numbers of examinees and items. Therefore, it can be used as a guide to practice.

#### Recovery of Ability Parameters

**Figure 6:**
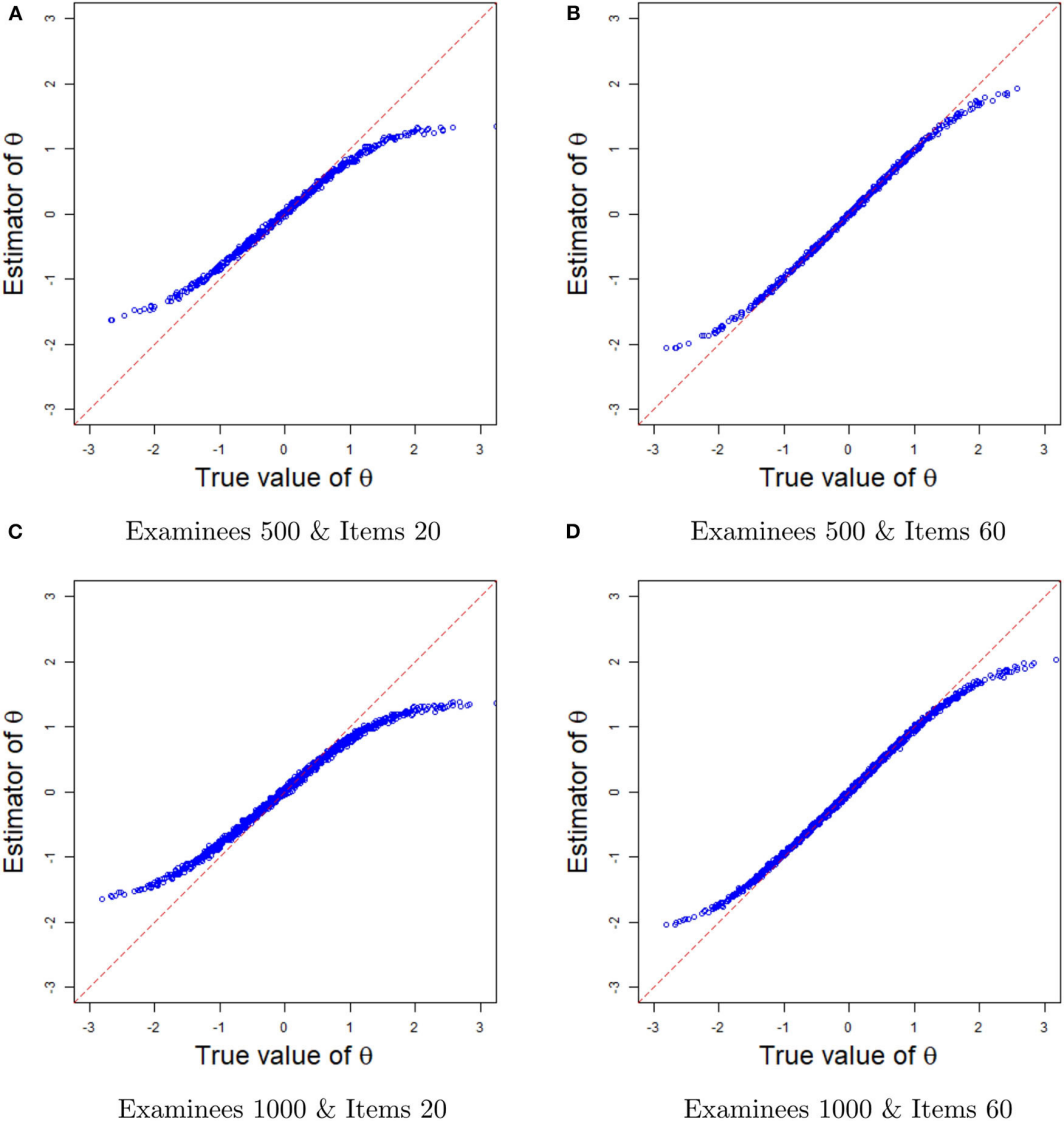
The comparisons between ability estimates and true values in different sample sizes. **(A)** The comparisons between ability estimates and true values based on 500 examinees and 20 items. **(B)** The comparisons between ability estimates and true values based on 500 examinees and 60 items. **(C)** The comparisons between ability estimates and true values based on 1,000 examinees and 20 items. **(D)** The comparisons between ability estimates and true values based on 1,000 examinees and 60 items.

Next, we evaluate the recovery of latent ability from the plots of the true values and the estimates in Figure 6. For a fixed number of examinees (500 or 1,000), when the number of items increases from 20 to 60, the ability estimates become more accurate, with the true values and the estimates basically lying on the diagonal line. Note that the estimated abilities are the average of 200 replication estimates. Because of the increase in the number of items, the probability of the situation in which all items are answered correctly by the high-ability examinees and incorrectly by the low-ability examinees, leading to a large deviation of the ability estimators, is reduced. Therefore, the estimated values and the true values of the ability at the end of the curve are closer to the diagonal line when the number of items is 60. In summary, the Bayesian sampling algorithm also provides accurate estimates of the ability parameters in term of the plots of the true values and the estimates.

### 4.2. Simulation 2

In this simulation study, we use the DIC and LPML model assessment criteria to evaluate model fitting. Two issues need further study. The first is whether the two criteria can accurately identify the true model that generates data from numerous fitting models. The second concerns the influence of different time weights in the G3PLT model on model fitting.

#### Simulation Design

In this simulation, the number of examinees is *N* = 1, 000 and the test length is fixed at 20. Six item response models will be considered: the traditional 3PL model and the G3PLT model with time weights *W* = 0, 2, 4, 6, and 8. Thus, we evaluate the model fitting in the following five cases:

- Case 1. True model: G3PLT model with time weight 0 vs. Fitted model: 3PL model, G3PLT model with time weight 0.
- Case 2. True model: G3PLT model with time weight 2 vs. Fitted model: 3PL model, G3PLT model with time weight 2.
- Case 3. True model: G3PLT model with time weight 4 vs. Fitted model: 3PL model, G3PLT model with time weight 4.
- Case 4. True model: G3PLT model with time weight 6 vs. Fitted model: 3PL model, G3PLT model with time weight 6.
- Case 5. True model: G3PLT model with time weight 8 vs. Fitted model: 3PL model, G3PLT model with time weight 8.

The true values and prior distributions for the parameters are the same as in Simulation 1. To implement the MCMC sampling algorithm, chains of length 10,000 with an initial burn-in period 5,000 are chosen. The results of Bayesian model assessment based on the 200 replications are shown in Table 4. Note that the following results for DIC and LPML are based on the average of 200 replications.

**Table 4:** The results of Bayesian model assessment in Simulation 2.

	**Fitted model**			**3PL**	**G3PLT0**	**G3PLT2**	**G3PLT4**	**G3PLT6**	**G3PLT8**
			*Q*_1_	25297.63	**25270.07**	–	–	–	–
		DIC	Median	25358.15	**25324.43**	–	–	–	–
			*Q*_3_	25412.52	**25379.70**	–	–	–	–
			IQR	114.88	**109.63**	–	–	–	–
	G3PLT0		*Q*_1_	−13456.37	**−13251.64**	–	–	–	–
		LPML	Median	−13431.01	**−13231.77**	–	–	–	–
			*Q*_3_	−13406.19	**−13218.86**	–	–	–	–
			IQR	50.17	**32.77**	–	–	–	–
			*Q*_1_	22742.44	–	**22677.65**	–	–	–
		DIC	Median	22851.46	–	**22777.38**	–	–	–
			*Q*_3_	22953.34	–	**22890.79**	–	–	–
	G3PLT2		IQR	210.89	–	**213.14**	–	–	–
			*Q*_1_	−12274.46	–	**−12246.10**	–	–	–
		LPML	Median	−12243.68	–	**−12221.93**	–	–	–
			*Q*_3_	−12221.43	–	**−12200.33**	–	–	–
			IQR	53.02	–	**45.76**	–	–	–
			*Q*_1_	20529.71	–	–	**20522.24**	–	–
		DIC	Median	20614.41	–	–	**20613.60**	–	–
			*Q*_3_	20711.15	–	–	**20708.31**	–	–
True			IQR	181.44	–	–	**186.06**	–	–
Model	G3PLT4		*Q*_1_	−11322.69	–	–	**−11263.87**	–	–
		LPML	Median	−11300.75	–	–	**−11239.84**	–	–
			*Q*_3_	−11273.01	–	–	**−11219.60**	–	–
			IQR	49.67	–	–	**44.26**	–	–
			*Q*_1_	20210.35	–	–	–	**20206.43**	–
		DIC	Median	20295.34	–	–	–	**20294.27**	–
			*Q*_3_	20386.09	–	–	–	**20384.67**	–
			IQR	175.73	–	–	–	**178.23**	–
	G3PLT6		*Q*_1_	−11102.84	–	–	–	**−11144.73**	–
		LPML	Median	−11079.08	–	–	–	**−11121.81**	–
			*Q*_3_	−11052.10	–	–	–	**−11098.77**	–
			IQR	50.74	–	–	–	**45.96**	–
			*Q*_1_	20014.40	–	–	–	–	**20013.64**
		DIC	Median	20111.34	–	–	–	–	**20112.86**
			*Q*_3_	20191.08	–	–	–	–	**20189.52**
			IQR	176.68	–	–	–	–	**175.87**
	G3PLT8		*Q*_1_	−11083.24	–	–	–	–	**−11032.39**
		LPML	Median	−11053.93	–	–	–	–	**−11007.48**
			*Q*_3_	−11026.44	–	–	–	–	**−10981.35**
			IQR	56.79	–	–	–	–	**51.03**

*Note that the 3PL denotes three parameter logistic model, and the G3PLTw denotes the general three parameter logistic model with time weight w, where w = 0, 2, 4, 6, 8*.

From Table 4, we find that when the G3PLT model with time weight 0 (G3PLT0) is the true model, the G3PLT0 model is chosen as the better-fitting model according to the results for DIC and LPML, which is what we expect to see. The medians of DIC and LPML are respectively 25 324.43 and −13231.77. The differences between the G3PLT0 model and 3PL model in the medians of DIC and LPML are −33.72 and 199.23, respectively. Similarly, when the G3PLT model with time weight 2 (G3PLT2) is the true model, the G3PLT2 model is also chosen as the better-fitting model according to the results for DIC and LPML. The medians of DIC and LPML are respectively 22 777.38 and −12221.93. The differences between the G3PLT2 model and 3PL model in the medians of DIC and LPML are −74.07 and 21.75, respectively. However, when the time weight increases from 4 to 8, the medians of DIC for the 3PL model and G3PLT model are basically the same. This shows that the 3PL model is basically the same as the G3PLT model with time weights 4, 6, and 8, which is attributed to the fact that the G3PLT model reduces to the traditional 3PL model when the time weight increases from 4 to 8. Based on the results for LPML, we find that the power of LPML to distinguish between the true G3PLT4 (6, 8) model and the 3PL model is stronger than that of DIC, because the LPMLs of the two models differ greatly. For example, the difference between the G3PLT8 model and 3PL model in the median of LPML is 46.45.

In summary, the two Bayesian model assessment criteria can accurately identify the true model that generates data. In addition, the process of transformation of the G3PLT model into the traditional 3PL model is also reflected by the differences in DIC and LPML. Therefore, the two Bayesian model assessment criteria are effective and robust and can guide practice.

## 5. Real Data

### 5.1. Data Description

**Figure 7:**
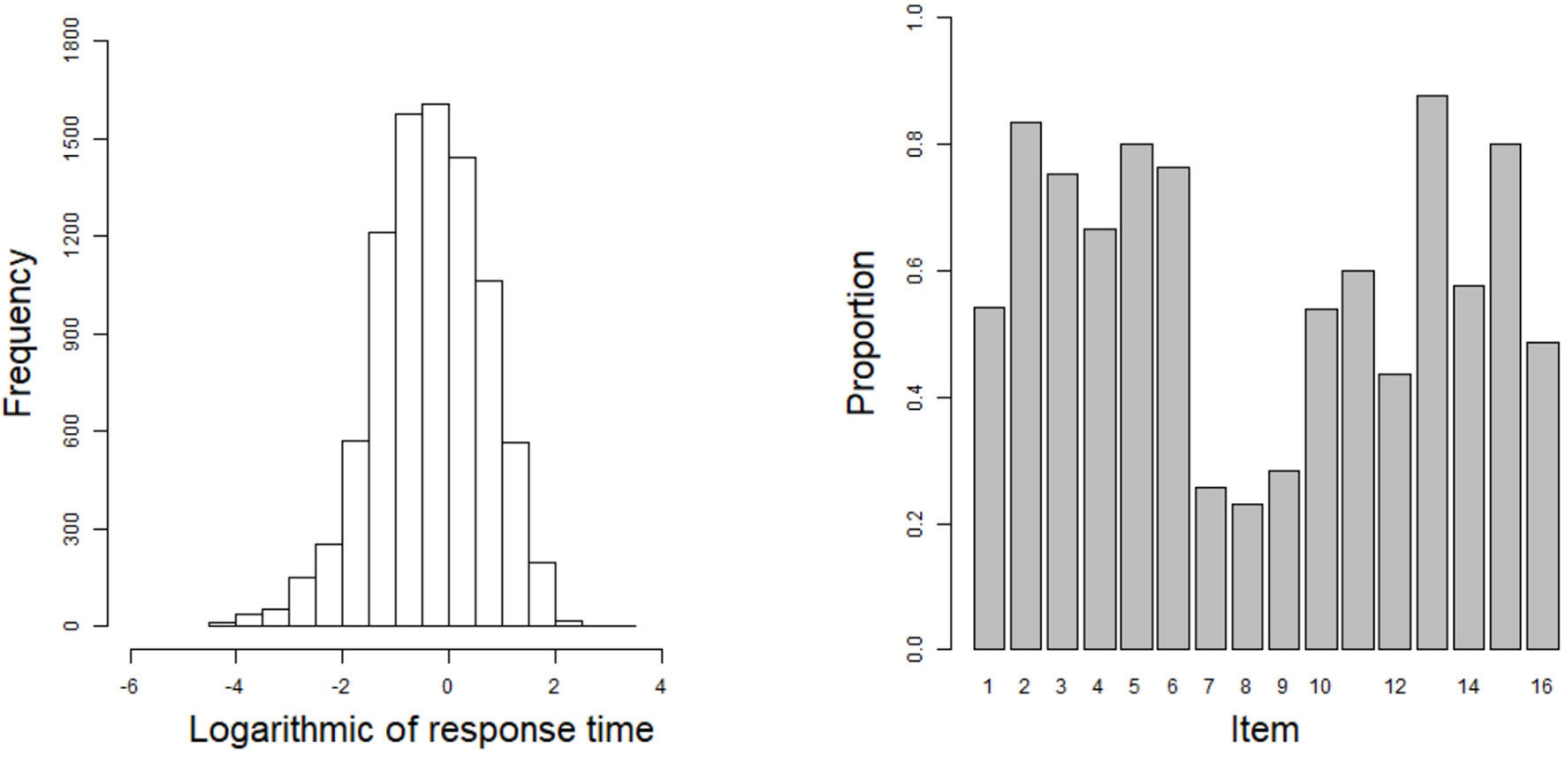
The frequency histogram of logarithmic response times and the correct rate for each item in the real data.

In this example, the 2015 computer-based Program for International Student Assessment (PISA) science data are used. From among the many countries that have participated in the computer-based assessment of the sciences, we choose the students from the USA as the object of analysis. Students with Not Reached (original code 6) or Not Response (original code 9) are removed in this study, where Not Reached and Not Response (omitted) are treated as missing data. The final 548 students are used to answer 16 items, and the corresponding response times are recorded. All 16 items are scored using a dichotomous scale. The 16 items are respectively CR083Q01S, CR083Q02S, CR083Q03S, CR083Q04S, DR442Q02C, DR442Q03C, DR442Q05C, DR442Q06C, CR442Q07S, CR245Q01S, CR245Q02S, CR101Q01S, CR101Q02S, CR101Q03S, CR101Q04S, and CR101Q05S. The frequency histogram of logarithmic response times and the correct rate for each item are shown in Figure 7.

### 5.2. Bayesian Model Assessment

To evaluate the impact of different time weights on the PISA data and to analyze the differences between the G3PLT model and the traditional 3PL model in fitting the data, both models are used to fit the data. G3PLT models with different time weights *W* = 0, 1, 2, 3, 4, 5, 6, 7, and 8 are considered. In the estimation procedure, the setting of the prior distributions is the same as in Simulation 1. In all of the Bayesian computations, we use 10,000 MCMC samples after a burn-in of 5,000 iterations for each model to compute all posterior estimates.

Table 5 shows the results for DIC and LPML under the 3PL model and the G3PLT model with different time weights. According to DIC and LPML, we find that the G3PLT model with time weight 6 is the best-fitting model, with DIC and LPML values of 8389.316 and −4196.672, respectively. The G3PLT model with time weight 0 is the worst-fitting model, with DIC and LPML values of 9708.940 and −4792.301, respectively. That the G3PLT model with time weight 0 is the worst fitting model can be attributed to the fact that the influence of the time effect on the correct-response probability is relatively weak for the PISA data. This is consistent with the the evaluation purpose of the PISA test, which is a nonselective and low-stakes test. Examinees lack motivation to answer each item carefully, and therefore the time effect cannot be reflected. However, when the time weight of the G3PLT model increases from 5 to 8, the DIC and LPML values are basically the same as those in the case of the 3PL model. The model fitting results once again verify that our G3PLT model reduces to the traditional 3PL model when the time weight increases to a certain value. Next, we will analyze the PISA data based on the G3PLT model with time weight 6.

**Table 5:** The results of Bayesian model assessment in real data analysis.

	**3PL**	**G3PLT0**	**G3PLT1**	**G3PLT2**	**G3PLT3**
DIC	8392.374	9708.940	9217.431	8825.986	8561.295
LPML	−4197.832	−4792.301	−4565.769	−4395.082	−4275.351
	**G3PLT4**	**G3PLT5**	**G3PLT6**	**G3PLT7**	**G3PLT8**
DIC	8441.556	8398.835	8389.316	8391.581	8390.254
LPML	−4221.003	−4200.857	−4196.672	−4197.678	−4197.906

### 5.3. Analysis of Item Parameters

The estimated results for the item parameters are shown in Table 6. We can see that the expected a posteriori (EAP) estimates of the nine item discrimination parameters are greater than one. This indicates that these items can distinguish well between different abilities. In addition, the EAP estimates of the 11 difficulty parameters are less than zero, which indicates that 10 items are slightly easier than the other six. The three most difficult items are items 8 (DR442Q06C), 7 (DR442Q05C), and 9 (CR442Q07S). The EAP estimates of the difficulty parameters for these three items are, respectively 1.085, 0.900, and 0.839. The corresponding correct rates for the three items in Figure 7 are 0.231, 0.257, and 0.285. The most difficult three items have the lowest correct rates, which is consistent with our intuition. The six EAP estimates of the guessing parameters are larger than 0.1. The three items that the examinees are most likely to answer correctly by guessing are items 11 (CR245Q02S), 12 (CR101Q01S), and 10 (CR245Q01S). The EAP estimates of the guessing parameters for these three items are respectively 0.132, 0.128, and 0.117. Among the 16 items, item 7 is the best design item owing to the fact that it has high discrimination and difficulty estimates, and the guessing parameter has the lowest estimate in all of the items. Next, we use the posterior standard deviation (SD) to evaluate the degree of deviation from the EAP estimate. The average SD of all discrimination parameters is about 0.005, the average SD of all difficulty parameters is about 0.010, and the average SD of all guessing parameters is about 0.001. We can see that the average SD values of the three parameters are very small, indicating that the estimated values fluctuate near the posterior mean.

**Table 6:** The results of item parameter estimation in real data analysis.

**Parameter**	**EAP**	**SD**	**HPDI**	**Parameter**	**EAP**	**SD**	**HPDI**
*a*_1_	0.980	0.003	[0.873, 1.120]	*a*_9_	1.199	0.003	[1.116, 1.312]
*a*_2_	0.927	0.003	[0.824, 1.025]	*a*_10_	0.821	0.004	[0.688, 0.946]
*a*_3_	0.986	0.004	[0.857, 1.114]	*a*_11_	1.059	0.006	[0.890, 1.200]
*a*_4_	1.034	0.003	[0.928, 1.139]	*a*_12_	1.004	0.007	[0.874, 1.195]
*a*_5_	0.893	0.007	[0.723, 1.047]	*a*_13_	1.037	0.006	[0.899, 1.198]
*a*_6_	1.084	0.005	[0.965, 1.211]	*a*_14_	1.011	0.005	[0.883, 1.137]
*a*_7_	1.216	0.005	[1.062, 1.336]	*a*_15_	0.986	0.006	[0.848, 1.190]
*a*_8_	1.087	0.004	[0.974, 1.203]	*a*_16_	0.803	0.002	[0.715, 0.917]
*b*_1_	−0.065	0.009	[−0.240, 0.111]	*b*_9_	0.839	0.007	[0.670, 0.995]
*b*_2_	−1.405	0.014	[−1.617, −1.170]	*b*_10_	0.065	0.020	[−0.186, 0.391]
*b*_3_	−0.921	0.010	[−1.085, −0.693]	*b*_11_	−0.147	0.016	[−0.374, 0.114]
*b*_4_	−0.519	0.009	[−0.700, −0.321]	*b*_12_	0.530	0.014	[0.324, 0.795]
*b*_5_	−1.187	0.021	[−1.430, −0.849]	*b*_13_	−1.608	0.015	[−1.846, −1.369]
*b*_6_	−0.920	0.011	[−1.124, −0.730]	*b*_14_	−0.083	0.012	[−0.280, 0.149]
*b*_7_	0.900	0.007	[0.726, 1.069]	*b*_15_	−1.145	0.016	[−1.429, −0.933]
*b*_8_	1.085	0.007	[0.876, 1.236]	*b*_16_	0.272	0.016	[0.062, 0.547]
*c*_1_	0.065	0.000	[0.018, 0.120]	*c*_9_	0.042	0.000	[0.016, 0.069]
*c*_2_	0.098	0.001	[0.026, 0.189]	*c*_10_	0.117	0.001	[0.029, 0.192]
*c*_3_	0.079	0.001	[0.017, 0.156]	*c*_11_	0.132	0.001	[0.056, 0.216]
*c*_4_	0.079	0.001	[0.015, 0.143]	*c*_12_	0.128	0.001	[0.071, 0.190]
*c*_5_	0.107	0.002	[0.028, 0.199]	*c*_13_	0.093	0.001	[0.028, 0.176]
*c*_6_	0.092	0.001	[0.019, 0.158]	*c*_14_	0.115	0.001	[0.034, 0.177]
*c*_7_	0.026	0.000	[0.006, 0.045]	*c*_15_	0.097	0.002	[0.022, 0.185]
*c*_8_	0.032	0.000	[0.009, 0.056]	*c*_16_	0.103	0.001	[0.035, 0.165]

### 5.4. Analysis of Personal Parameters

Next, we analyze the differences between the estimated abilities of examinees in the 3PL model and in the G3PLT model under the same response framework, together with the reasons for these differences. We consider four examinees with same response framework for the 16 items, (1, 1, 1, 1, 1, 1, 1, 0, 1, 1, 1, 1, 1, 1, 1, 1). They are examinee 60, examinee 313, examinee 498, and examinee 210, and the corresponding response times for these examinees to answer the 16 items are 25.80, 29.36, 35.48, and 41.44 min. Under the framework of the 3PL model, the estimated abilities of the four examinees are the same, 1.45. However, taking into account the time factors for the four examinees, the estimated abilities are different according to the G3PLT model with time weight 6. The estimated abilities are 1.46, 1.42, 1.41, and 1.38, respectively. We find that under the same response framework, as the response times of the examinees increase from 25.80 to 41.44 min, the estimated abilities of the examinees show a decreasing trend. This indicates that examinees with short response times are more proficient in answering these items than examinees with long response times. Therefore, the ability of examinees with short response times to answer 15 items correctly should be higher than that of examinees with long times. This once again shows that our G3PLT model is reasonable. By incorporating the time effect into the IRT model, the interpretation of the latent construct essentially shifts: before we were measuring whether students could answer items correctly, now we are measuring whether students can answer items correctly and quickly.

## 6. Concluding Remarks

In this paper, we propose a new and flexible general three-parameter logistic model with response time (G3PLT), which is different from previous response time models, such as the hierarchical model framework proposed by van der Linden (2007), in which the response and the response time are considered in different measurement models, while a high-level model represents the correlation between latent ability and speed through a population distribution. However, our model integrates latent ability, time, and item difficulty into a item response model to comprehensively consider the impact on the probability of correct response. This approach to modeling is simpler and more intuitive. In addition, time weights are introduced in our model to investigate the influence of time intensity limited by different tests on the correct-response probability. When the time weight reaches 8, our model reduces to the traditional 3PL model, which indicates that the time factor has little influence on the correct-response probability. The examinees then answer each item correctly with the response probability given by the 3PL model.

However, the computational burden of the Bayesian algorithm becomes excessive when large numbers of examinees or items are considered or a large MCMC sample size is used. Therefore, it is desirable to develop a standalone R package associated with C++ or Fortran software for more extensive large-scale assessment programs.

Other issues should be investigated in the future. First of these is whether the G3PLT model can be combined with a multilevel structure model to analyze the influence of covariates on the latent ability at different levels, for example, to explore the influence of the time effect, gender, and socioeconomic status on latent ability. Second, although we have found that for different examinees with the same response framework, the ability estimates from the 3PL model is the same, those from the G3PLT model differ greatly. Examinees who take less time should be more proficient in answering items, and their ability should be higher than that of examinees who take longer. “Proficiency” is a latent skill that is not the same as latent ability. Whether we can connect proficiency and latent ability through a multidimensional 3PLT model to analyze their relationship is also an important topic for our future research. Third, our new model can also be used to detect various abnormal response behaviors, such as rapid guessing and cheating, with the aim of eliminating deviations in ability estimates caused by such behaviors.

## Data Availability Statement

Publicly available datasets were analyzed in this study. This data can be found here: http://www.oecd.org/pisa/data/.

## Author Contributions

All authors listed have made a substantial, direct and intellectual contribution to the work, and approved it for publication.

## Conflict of Interest

The authors declare that the research was conducted in the absence of any commercial or financial relationships that could be construed as a potential conflict of interest.
